# The impact of comorbidities on productivity loss in asthma patients

**DOI:** 10.1186/s12931-016-0421-9

**Published:** 2016-08-26

**Authors:** Solmaz Ehteshami-Afshar, J. Mark FitzGerald, Christopher Carlsten, Hamid Tavakoli, Roxanne Rousseau, Wan Cheng Tan, J. Douglass Rolf, Mohsen Sadatsafavi

**Affiliations:** 1 Experimental Medicine Program, Department of Medicine, Faculty of Medicine, The University of British Columbia, Vancouver, Canada; 2 Department of Medicine, Division of Respiratory Medicine, The University of British Columbia, Vancouver, Canada; 3 Institute for HEART + LUNG Health, Department of Medicine (Respiratory Division), The University of British Columbia, Vancouver, Canada; 4 Centre for Clinical Epidemiology and Evaluation, The University of British Columbia, Vancouver, Canada; 5 Department of Medicine, Centre for Occupational and Environmental Lung Disease, Vancouver, BC Canada; 6 Centre for Heart Lung Innovation, The University of British Columbia, Vancouver, Canada; 7 Kelowna Allergy & Respirology Research, Kelowna, Canada; 8 Institute of Heart and Lung Health, The Lung Centre, 2775 Laurel Street, Vancouver, BC V5Z 1 M9 Canada

**Keywords:** Asthma, Comorbidities, Productivity loss, Presenteeism, Absenteeism

## Abstract

**Background:**

Health-related productivity loss is an important, yet overlooked, component of the economic burden of disease in asthma patients of a working age. We aimed at evaluating the effect of comorbidities on productivity loss among adult asthma patients.

**Methods:**

In a random sample of employed adults with asthma, we measured comorbidities using a validated self-administered comorbidity questionnaire (SCQ), as well as productivity loss, including absenteeism and presenteeism, using validated instruments. Productivity loss was measured in 2010 Canadian dollars ($). We used a two-part regression model to estimate the adjusted difference of productivity loss across levels of comorbidity, controlling for potential confounding variables.

**Results:**

284 adults with the mean age of 47.8 (SD 11.8) were included (68 % women). The mean SCQ score was 2.47 (SD 2.97, range 0–15) and the average productivity loss was $317.5 per week (SD $858.8). One-unit increase in the SCQ score was associated with 14 % (95 % CI 1.02–1.28) increase in the odds of reporting productivity loss, and 9.0 % (95 % CI 1.01–1.18) increase in productivity loss among those reported any loss of productivity. A person with a SCQ score of 15 had almost $1000 per week more productivity loss than a patient with a SCQ of zero.

**Conclusions:**

Our study deepens the evidence-base on the burden of asthma, by demonstrating that comorbidities substantially decrease productivity in working asthma patients. Asthma management strategies must be cognizant of the role of comorbidities to properly incorporate the effect of comorbidity and productivity loss in estimating the benefit of disease management strategies.

## Background

With increasing life expectancy there has been an increase in the prevalence of many chronic diseases and the co-existence of multiple diseases [1–3]. Clinically, comorbidities are relevant given their potential effect on the index disease in terms of diagnosis, prognosis, and management [4]. Also, comorbid conditions increase the need for medication, risk of adverse effects and drug interactions, and reduce adherence to treatments, quality of life and functional status [1, 5, 6]. Patients with multiple comorbidities tend to use more medical services and impose a greater burden on the health-care system [2, 6].

Asthma is associated with several comorbidities; however, the prevalence varies across studies [5–8]. In a study from the United States, 26 and 10 % of asthma patients had at least one or ≥3 comorbidities, respectively [7]. In a study from Germany, 26 % of asthma patients had at least one other comorbidity while 17 % had 2 or more [9]. In a Canadian study, almost 60 % of asthma patients had at least one comorbidity [6], while in another study 12.5 % of adult asthma patients reported having three or more comorbidities, increasing to 20 % for adults 55 years and older [5, 6].

It has been well demonstrated that comorbidities are associated with poor outcomes in asthma patients [10]. Asthma patients with comorbidities experience more asthma exacerbations [6, 11–13] and there is a significant relationship between asthma control and the presence of comorbidities [4, 14, 15]. The reason behind this fact is unclear. It could be because the patient places a higher priority on other health conditions, which influences the adherence to asthma treatment. Also the nature of comorbidities like depression may cause the patient to pay less attention to their general health status and care less [7]. In a Canadian province, British Columbia (BC), 25 % of asthma patients have depression [5]. Also, comobidities could directly and causally affect the severity of asthma or its responsiveness to treatment; examples include rhinitis and gastroesophageal reflux disease [16].

It has been demonstrated that indirect costs of asthma accounted for the greater proportion of costs of asthma than direct costs, however most of the studies unnoticed this amount [16]. Also despite the documented burden of comorbidities in asthma, their effect on productivity loss has been overlooked in the past. One reason behind this status is that asthma patients are a relatively young population and are assumed to be free of comorbidities [15]. The general increase in longevity and the increase in the retirement age will inevitably result in more and more working asthma subjects. The aim of the present study was to evaluate the effect of comorbidities on productivity loss in a population-based sample of adult asthma patients.

## Methods

### Study design and participants

This study is based on data from the Economic Burden of Asthma (EBA), a 1-year prospective cohort study with the specific aim of estimating the economic and humanistic burden of asthma (University of British Columbia Human Ethics Board H10-01542). In the EBA study, 618 patients with self-reported physician diagnosis asthma who were aged 1–85 were recruited by random digit dialing and followed up for a year. The study’s catchment areas were Vancouver and Central Okanagan census areas, the latter being in the interior of the BC Province with a large fraction of the population residing in rural areas. The details of this study have been described previously [17, 18] and the inclusion criteria of the present study are the same as the main EBA, having at least one encounter with healthcare system because of asthma in the past 5 years and having no plan to move out of the region in the next year, except it was restricted to adult (≥19 years old) patients who were employed at the baseline visit.

### Variables

#### Comorbidity

A comorbidity score was calculated based on the Self-administered Comorbidity Questionnaire (SCQ) administered in the last visit [19]. The recall period of the questionnaire is 12 months and thus we assumed comorbidity score was constant across the study period [19]. The SCQ score not only considers the number of the comorbidities but also their severity. Each included comorbid condition can get a maximum of three points based on the presence of disease, whether receiving treatment, and any functional limitation due to the condition. This questionnaire has been validated and has a moderately strong correlation with the widely popular Charlson comorbidity index [19]. The Charlson index is mainly designed for hospitalized patients and its evaluation needs access to medical records [19]. On the other hand, the SCQ is designed and validated for the outpatient settings by relying on patient self report as the principle source of information [19]. The original SCQ includes 13 common comorbidities, but in this study the questions related to pulmonary disorders were excluded (given that all patients had asthma), leaving the questionnaire with a maximum of 36 scores, three points for each of the 12 questions. The included comorbidities were heart disease, hypertension, diabetes mellitus, ulcer or stomach disease, kidney disease, liver disease, anemia or other blood disease, cancer, depression, osteoarthritis or degenerative arthritis, back pain, and rheumatoid arthritis.

#### Productivity loss

Productivity loss was measured at baseline by two validated questionnaires: the Work Productivity and Activity Impairment (WPAI) [20], and the Valuation of Lost Productivity (VOLP) [21]. The WPAI records patients’ absenteeism (missing work due to health conditions) and presenteeism (attending work but not being fully functional) in the last 7 days by asking about the hours they missed from work because of sick days or the times they went in late or left early due to health status and times they were not functional with limited accomplishment and unable to concentrate on their tasks due to the health status respectively [20]. The VOLP questionnaire collects information about the work environment such as time sensitivity of the job, team work, and availability of replacement, to calculate a coefficient that measures the contribution of individual to the work place (a coefficient of X indicates that each hour of a person’s absence is equivalent of X hours of work loss) [21, 22].

The monetary value of productivity loss per week was the product of three terms: amount of work time (hours) lost, the VOLP coefficient, and the hourly wage of the individual. Job titles were matched to the National Occupation Classification (NOC) codes [23] to estimate the hourly wage based on sex and age for each NOC from Statistics Canada for year 2010 [17]. The reported weekly costs are therefore in 2010 Canadian dollars (CAD).

### Confounders

Socio-demographic data collected at the baseline visit were included in the statistical models as potential confounders (factors that can affect both comorbid level and productivity but are not on the causal pathway). They included: sex, age, household income levels (low v. high at cut-off of CAD$60,000 per year), education (low v. high at cut-off of 4-year college/university degree), type of residence (urban v. rural), place of birth (Canada v. abroad), drug insurance (having no insurance, being partially insured, or being fully insured), and the proportion of days covered (PDC) by any asthma controller medication in past 12 months as an indicator of adherence (cut-off values <50 %, 50–80 %, ≥80 %).

The main analysis did not adjust for asthma control as it cannot be a confounder; rather, it is potentially being on the causal pathway (that is, part of the impact of comorbidity on productivity might be due to the comorbid conditions’ affecting the likelihood of achieving asthma control. It is also very unlikely for the current asthma control status to have an effect on comorbidities (thus being confounding factor). But a sensitivity analysis was performed to assess the effect of adjusting for control status on the outcomes. We defined control status based on Global Initiative for Asthma (GINA) 2012 definition, which included the presence of symptoms and impairment in lung function (all measured at baseline visit).

### Statistical analysis

All analyses were performed using Stata (version 14; StataCorp, College Station, TX, USA). Two-tailed *p*-values at 0.05 were considered statistically significant. Descriptive analysis was performed on the baseline variables. We reported the hours and costs of both components of productivity loss (absenteeism and presenteeism), as well as total productivity loss across different levels of SCQ score.

As the productivity loss data were zero-inflated, we used two-part models for statistical inference [24]. The first part was a logistic component and the second part was a generalized linear model with logarithmic link function and gamma distribution. The first part generates odds ratio (OR) associating covariates with any loss of productivity, and the second component produces relative rate (RR) associating covariates with the magnitude of productivity loss among those with any loss of productivity. For both components the dependent variable was the monetary value of productivity loss and the independent variables were the SCQ score and other covariates as previously mentioned. As there were missing values among some of the covariates, multiple imputations were first performed, creating 5 imputed datasets without missing variables; results of separate analyses on the imputed datasets were combined. To estimate the marginal effect of SCQ on productivity loss (that is, the weekly loss of productivity associated with any level of SCQ score), the OR and RR from the two components were combined, and *p*-values and confidence intervals were estimated using bootstrapping (500 times) as described elsewhere [25]. The procedure was conducted separately with absenteeism, presenteeism, and total productivity loss as the dependent variable.

## Results

**Fig. 1:**
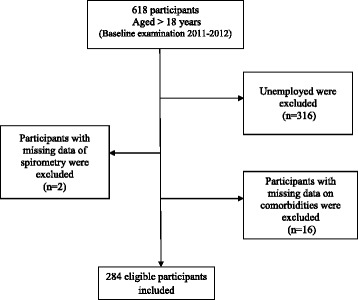
Flow chart of study population

Figure 1 shows the flowchart of sample selection. The final sample consisted of 284 individuals whose baseline characteristics are shown in Table 1. The sample was 68 % female with a mean age of 47.8 ± 11.8 with generally high levels of education and household income. Most of the subjects (63 %) had at least one comorbid condition and the overall SCQ score was 2.47 ± 2.97, with a minimum of 0 and a maximum of 15. Only 48 % of patients reported any productivity loss, with 36 % of them reporting absenteeism and 64 % reporting presenteeism. Mean weekly hours and costs of productivity loss were 16 ± 17.6 h and $317.49 ± $858.83 respectively.

**Table 1:** Characteristics of study sample

	Study population = 284
Age, mean ± SD^a^	47.8 ± 11.8
Sex (%)	
Women	193 (68)
Men	91 (32)
Household income (%)	
High (>60,000 CAD)	178 (62.7)
Low	96 (33.8)
Missing	10 (3.5)
Educational level (%)	
High	229 (80.6)
Low	55 (19.4)
Place of birth (%)	
Canada	207 (72.9)
Outside Canada	77 (27.1)
Ethnicity (%)	
Caucasian	231 (81.3)
Asian	18 (6.3)
Other	35 (12.4)
Residence type (%)	
Urban	260 (91.5)
Rural	24 (8.5)
Asthma medication adherence (%)	
PDC^b^ < 50 %	171 (60.2)
50 % ≤ PDC < 80 %	31 (11)
PDC ≥ 80 %	81 (28.5)
Missing	1 (0.3)
Asthma control level (%)	
Controlled	55 (19.4)
Partially Controlled	113 (39.8)
Uncontrolled	115 (40.5)
Missing	1 (0.3)
Productivity Loss (%)	136 (48)
Absenteeism (%)	49 (17)
Presenteeism (%)	127 (45)
Hours of overall productivity loss, mean ± SD	16 ± 17.6
Costs^c^ of overall productivity loss, mean ± SD	317.49$ ± 858.83$
Overall SCQ^d^ comorbidities score, mean ± SD	2.47 ± 2.97
Heart disease (%)	15 (5.3)
Hypertension (%)	35 (12.3)
Diabetes (%)	9 (3.2)
Ulcer or Stomach Disease (%)	37 (13)
Kidney disease (%)	3 (1.1)
Liver disease (%)	2 (0.7)
Anemia or other blood disease (%)	21 (7.4)
Cancer (%)	6 (2.1)
Depression (%)	40 (14.1)
Osteoarthritis, degenerative arthritis (%)	62 (21.8)
Back pain (%)	99 (34.9)
Rheumatoid arthritis (%)	2 (0.7)

^a^Standard deviation

^b^proportions of days covered

^c^2010 Canadian dollars

^d^self-administered comorbidity questionnaire

### Unadjusted analysis

Table 2 shows the results of unadjusted analysis. The hours of absenteeism increased from 1.26 to 7.14 h as the SCQ increased from 0 to 15, and for presenteeism it rose from 3.97 to 12.59 h. The costs of absenteeism increased from $50/week for SCQ of 0 to almost $300/week for SCQ of 15, while the corresponding values for presenteeism was $140/week and $734/week. The same increases were seen for the total productivity loss, from $190/per week to $1036/per week.

**Table 2:** Unadjusted regression analysis

SCQ score	Hours of Absenteeism	Costs of Absenteeism^a^	Hours of Presenteeism	Costs of Presenteeism^a^	Hours of total productivity loss	Costs of productivity loss^a,b^
0	1.26	50.35	3.97	140.20	5.22	190.18
	(0.54–1.98)	(19.88–80.81)	(2.73–5.22)	(65.27–215-13)	(3.49–6.96)	(97.22–283.14)
1	1.65	66.91	4.55	179.81	6.20	246.60
	(0.99–2.32)	(40.19–93.63)	(3.48–5.62)	(118.75–240.87)	(4.70–7.70)	(169.16–324.03)
2	2.04	83.47	5.12	219.41	7.17	303.01
	(1.22–2.87)	(49.80–117.14)	(4.00–6.25)	(145.85–292.98)	(5.52–8.83)	(208.30–397.72)
3	2.44	100.03	5.70	259.02	8.15	359.43
	(1.33–3.54)	(53.27–146.79)	(4.31–7.08)	(155.75–362.28)	(6.04–10.26)	(226.90–491.95)
4	2.83	116.59	6.27	298.62	9.13	415.84
	(1.38–4.27)	(54.35–178.83)	(4.51–8.03)	(159.03–438.21)	(6.41–11.85)	(237.59–594.10)
5	3.22	133.15	6.84	338.22	10.10	472.26
	(1.41–5.03)	(54.45–211.86)	(4.65–9.04)	(159.70–516.76)	(6.70–13.50)	(245.10–699.42)
6	3.61	149.71	7.42	377.83	11.08	528.67
	(1.43–5.79)	(54.06–245-36)	(4.76–10.08)	(159.12–596.53)	(6.96–15.20)	(251.09–806.25)
7	4.00	166.27	7.99	417.43	12.05	585.09
	(1.44–6.56)	(53.42–279.13)	(4.85–11.13)	(157.90–676.97)	(7.19–16.91)	(256.28–913.90)
8	4.39	182.83	8.57	457.04	13.03	641.50
	(1.45–7.34)	(52.61–313.06)	(4.94–12.20)	(156.28–757.79)	(7.42–18.64)	(260.97–1022.03)
9	4.79	199.39	9.14	496.64	14.01	697.92
	(1.45–8.12)	(51.70–347.09)	(5.01–13.27)	(154.41–838.87)	(7.63–20.38)	(265.36–1130.48)
10	5.18	215.96	9.72	536.24	14.98	754.33
	(1.46–8.90)	(50.72–381.19)	(5.09–14.34)	(152.38–920.11)	(7.84–22.12)	(269.53–1239.14)
11	5.57	232.52	10.29	575.85	15.96	810.75
	(1.46–9.68)	(49.69–415.35)	(5.16–15.42)	(150.23–1001.47)	(8.05–23.86)	(273.56–1347.94)
12	5.96	249.08	10.86	615.45	16.93	867.17
	(1.46–10.46)	(48.62–449.54)	(5.23–16.50)	(147.98–1082.92)	(8.26–25.61)	(277.48–1456.86)
13	6.35	265.64	11.44	655.06	17.91	923.58
	(1.46–11.25)	(47.52–483.75)	(5.29–17.58)	(145.68–1164.43)	(8.46–27.36)	(281.31–1565.85)
14	6.74	282.20	12.01	694.66	18.88	979.99
	(1.45–12.03)	(46.41–517.99)	(5.36–18.67)	(143.32–1246)	(8.66–29.11)	(285.08–1674.91)
15	7.14	298.76	12.59	734.26	19.86	1036.41
	(1.45–12.82)	(45.27–552.25)	(5.42–19.75)	(140.93–1327.60)	(8.86–30.86)	(288.80–1784.03)

All the *p*-values <0.05

^a^2010 CAD

^b^The sum of the costs of absenteeism and presenteeism are not exactly equal to the costs of total productivity loss, because the exact distribution of error terms around each component is inevitably different in regression models

### Adjusted analysis

The results of two-part regression model are demonstrated in Table 3. In the logistic part of the analysis, SCQ was significantly associated with higher odds of reporting absenteeism, presenteeism and total productivity loss. However, in the second part of the regression, among patients with productivity loss, SCQ was only significantly associated with the total productivity loss (RR = 1.09, CI = 1.01-1.18, *P* = 0.02) and not presenteeism or absenteeism separately. The other covariates were not significantly associated with productivity loss in either parts of the model.

**Table 3:** Results of the adjusted regression analysis of productivity loss on SCQ^a^ score

		Absenteeism	Presenteeism	Total Productivity Loss
Female v. male	Adjusted OR for reporting productivity loss	1.72	1.08	0.99
		(0.53–2.57)	(0.61–2.35)	(0.56–1.76)
		(*P* = 0.7)	(*P* = 0.77)	(*P* = 0.99)
	Adjusted ratio of productivity loss among those who reported productivity loss	0.42	0.88	0.79
		(0.15–1.18)	(0.52–2.18)	(0.46–1.37)
		(*P* = 0.1)	(*P* = 0.63)	(*P* = 0.41)
Age (per 1 year increase)	Adjusted OR for reporting productivity loss	0.98	0.98	0.98
		(0.95–1.01)	(0.96–1.04)	(0.96–1.01)
		(*P* = 0.31)	*P* = (0.13)	(*P* = 0.23)
	Adjusted ratio of productivity loss among those who reported productivity loss	0.98	0.99	0.98
		(0.94–1.03)	(0.96–1.04)	(0.96–1.01)
		(*P* = 0.5)	(*P* = 0.49)	(*P* = 0.18)
High v. urban education	Adjusted OR for reporting productivity loss	1.16	0.68	0.52
		(0.41–3.27)	(0.34–2.84)	(0.25–1.1)
		(*P* = 0.73)	(*P* = 0.27)	(*P* = 0.09)
	Adjusted ratio of productivity loss among those who reported productivity loss	0.92	1.50	1.44
		(0.29–2.96)	(0.84–2.40)	(0.76–2.72)
		(*P* = 0.9)	(*P* = 0.17)	(*P* = 0.26)
Rural residence	Adjusted OR for reporting productivity loss	0.47	0.38	0.41
		(0.13–1.75)	(0.11–6.17)	(0.13–1.35)
		(*P* = 0.26)	(*P* = 0.11)	(*P* = 0.14)
	Adjusted ratio of productivity loss among those who reported productivity loss	0.99	0.42	0.44
		(0.05–20.88)	(0.21–2.88)	(0.16–1.15)
		(*P* = 0.99)	(*P* = 0.1)	(*P* = 0.1)
Foreign Born v. Canadian-born	Adjusted OR for reporting productivity loss	0.95	1.00	1.21
		(0.41–2.19))	(0.56–2.42)	(0.67–2.17)
		(*P* = 0.92)	(*P* = 0.99)	(*P* = 0.53)
	Adjusted ratio of productivity loss among those who reported productivity loss	0.41	0.84	0.72
		(0.12–1.39)	(0.52–2.08)	(0.43–1.19)
		(*P* = 0.16)	(*P* = 0.5)	(*P* = 0.2)
PDC^b^ Level (Reference: PDC < 50 %)				
50–80 %	Adjusted OR for reporting productivity loss	2.75	1.27	1.34
		(0.98–7.73)	(0.54–3.66)	(0.58–3.10)
		(*P* = 0.07)	(*P* = 0.59)	(*P* = 0.49)
	Adjusted ratio of productivity loss among those who reported productivity loss	1.49	2.50	2.72
		(0.33–6.64)	(0.70–6.77)	(0.91–8.10)
		(*P* = 0.6)	(*P* = 0.15)	(*P* = 0.07)
>80 %	Adjusted OR for reporting productivity loss	1.11	0.86	0.97
		(0.48–2.59)	(0.47–2.50)	(0.52–1.80)
		(*P* = 0.8)	(*P* = 0.6)	(*P* = 0.93)
	Adjusted ratio of productivity loss among those who reported productivity loss	0.95	1.13	1.13
		(0.40–2.29)	(0.69–2.08)	(0.68–1.86)
		(*P* = 0.91)	(*P* = 0.62)	(*P* = 0.64)
Drug Insurance (Reference: full insurance)				
Partial	Adjusted OR for reporting productivity loss	1.36	1.03	1.11
		(0.41–4.49)	(0.45–3.44)	(0.47–2.63)
		(*P* = 0.59)	(*P* = 0.94)	(*P* = 0.8)
	Adjusted ratio of productivity loss among those who reported productivity loss	0.84	0.60	0.73
		(0.20–3.53)	(0.24–3.99)	(0.30–1.80)
		(*P* = 0.82)	(*P* = 0.17)	(*P* = 0.5)
None	Adjusted OR for reporting productivity loss	0.82	0.91	0.86
		(0.20–3.43)	(0.36–4.07)	(0.33–2.25)
		(*P* = 0.82)	(*P* = 0.86)	(*P* = 0.76)
	Adjusted ratio of productivity loss among those who reported productivity loss	1.09	0.66	0.76
		(0.16–7.33)	(0.24–4.58)	(0.28–2.11)
		(*P* = 0.93)	(*P* = 0.42)	(*P* = 0.61)
High v. low income	Adjusted OR for reporting productivity loss	0.84	0.87	0.88
		(0.37–1.88)	(0.48–2.47)	(0.47–1.66)
		(*P* = 0.54)	(*P* = 0.66)	(*P* = 0.7)
	Adjusted ratio of productivity loss among those who reported productivity loss	1.47	1.27	1.27
		(0.55–3.93)	(0.74–2.25)	(0.69–2.32)
		(*P* = 0.44)	(*P* = 0.38)	(*P* = 0.44)
SCQ (per 1 unit increase)	Adjusted OR for reporting productivity loss	1.17	1.14	1.14
		(1.04–1.32)	(1.03–1.17)	(1.02–1.28)
		(*P* = 0.01)	(*P* = 0.01)	(*P* = 0.01)
	Adjusted ratio of productivity loss among those who reported productivity loss	1.04	1.05	1.09
		(0.90–1.21)	(0.98–1.11)	(1.01–1.18)
		(*P* = 0.56)	(*P* = 0.14)	(*P* = 0.02)

^a^Self-administered comorbidity questionnaire

^b^proportion of days covered by medication

### Marginal effect of comorbidity on productivity loss

**Fig. 2:**
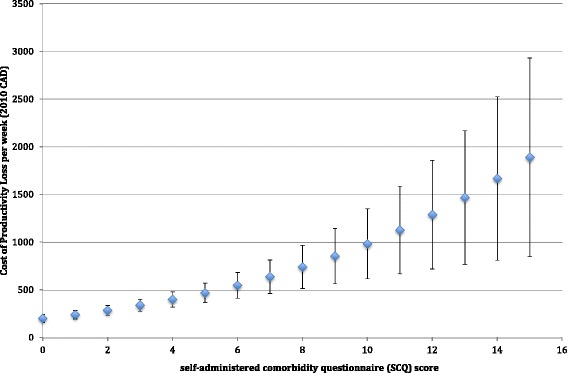
Incremental Costs of productivity loss based on comorbidity scores

The marginal effect of each level of SCQ score on total productivity loss is demonstrated in Fig. 2. In patients without any comorbidity, the productivity loss was $205 week. Total productivity loss was $1685 higher with a SCQ score of 15 in comparison to a SCQ score of zero. The margins were significant at all the levels, except SCQ score of 15 (*P*-value = 0.06). For Absenteeism, the costs were from $61.87/week (SD = 23.07) for SCQ score of 0 to $612.61/week (SD = 498.06) for those with the score of 15, and for presenteeism they were from $160.92/week (SD = 32.57) to $877.33/week (SD = 473) for the SCQ scores of 0 and 15, respectively. However the incremental costs for the SCQ score of ≥10 for absenteeism and SCQ score of 15 for presenteeism were not significant.

### Sensitivity analysis

Sensitivity analysis revealed that OR for reporting productivity loss and adjusted ratio for productivity loss among those reporting it did not change by adding control status in the model. However, the adjusted RR in the second part was no longer significant. Adding control status to the model did not have a significant impact on the estimates of the marginal loss of productivity (Appendix).

## Discussion

In this study, we have demonstrated that as the SCQ score, a validated quantitative measure of the burden of comorbidity, increased, the hours of absenteeism and presenteeism increased significantly to almost 20 h per week. This caused almost $1685/week higher productivity loss in patients with a score of 15, the maximum score observed in our sample, in comparison to those with a zero score. The average SCQ score in the sample was 2.47 (SD 2.97). At this level, productivity loss was almost 1.5 times higher than in individuals without any comorbidity (SCQ = 0). In the full two-part regression, SCQ increased the odds of reporting productivity loss, absenteeism and or presenteeism by 14–17 %. In addition, among those with productivity loss, one-unit increase in SCQ increased productivity loss by 9 %. Overall, our results demonstrate the substantial effect of comorbidity on productivity loss in patients with asthma.

Previous studies assessing the impact of comorbidities on asthma patients mostly focused on direct costs or health services use [7, 12, 15, 26]. For example, they have demonstrated that the rate of hospitalization due to asthma and Emergency Department (ED) visits in asthma patients increased in the presence of comorbidities [7, 12, 15, 26]. It has also been shown that the presence of some comorbidities increase the risk of mortality [10, 26]. The relationship between comorbidities and asthma exacerbations has also been demonstrated [6]. A study conducted in Finland showed that the presence of one and more than two comorbidities increased the risk of work disability with hazard ratios of 2.2 and 4.5, respectively [27]. In that study, work disability was defined as long-term sickness absence (≥90 days) and receiving a disability pension. Results of current study are inline with our previous study that demonstrated the presence of comorbid psychological conditions in asthma patients will increase productivity loss significantly [28].

To the best of our knowledge, there is no other study assessing the general impact of comorbidities on productivity loss in asthma patients including both absenteeism and presenteeism and transforming the productivity loss into its monetary value. The use of validated instruments enabled us to transform productivity loss time to its monetary value, incorporating the impact of the affected individual on team productivity, and the use of a robust statistical method enabled us to properly handle statistical issues around zero-inflated and skewed costs data.

Besides these strengths, our study has several limitations worth mentioning. First, the final sample size (284) might have underpowered the results and our sample were mostly highly educated with high income, which could manifest the healthy volunteer bias. Second, our sample only included employed asthma patients. None of the participants in the original study reported being unemployed because of asthma. As such, we could not incorporate the loss of productivity for asthma patients who lose their job due to the asthma-related or comorbidity-related impairment. Third, self-reported physician diagnosis of asthma and self-reported comorbidities and productivity loss might reduce the accuracy of the data we used. Fourth, the percentage of patients with higher scores of SCQ was limited such that the results for the patients with SCQ scores of ≥10 should be interpreted cautiously. Ultimately, the aspect of the burden of a disease that is the most relevant for clinical practice and policy-making is the component that can be prevented by disease management. Having documented a significant association between comorbidity and productivity loss, the research agenda should move forward to studying specific comorbid conditions as well as the impact of treatment on preventing such loss of productivity.

## Conclusions

Taking the limitations into account, our study has highlighted the important associations of comorbidities with productivity loss in working asthma patients. This is demonstrated by almost $1685/week higher productivity loss in patients with a SCQ score of 15 in comparison to those with a zero score. Productivity loss is a disregarded aspect of the economic burden of asthma [16]. Thus this study is a reminder for health care providers to pay greater attention to comorbidities in the management of asthma in order to reduce the burden of this common disease that disproportionately affects individuals in their productive years of life.

## Appendix

**Table 4:** Costs of productivity loss per week (CAD)^b^, comparing the main model with alternative model adding control status as confounder

SCQ score	Main Model	Alternative model^a^
0	205.12 ± 42.09	215.05 ± 50.51
1	244.42 ± 45.46	253.10 ± 53.85
2	290.07 ± 51.73	296.62 ± 59.65
3	342.81 ± 62.54	346.31 ± 69.40
4	403.47 ± 79.29	402.74 ± 84.45
5	472.90 ± 103.02	466.56 ± 105.91
6	552.02 ± 134.70	538.45 ± 134.69
7	641.82 ± 175.39	619.12 ± 171.69
8	743.35 ± 226.39	709.36 ± 217.94
9	857.74 ± 289.24	809.96 ± 274.69
10	986.20 ± 365.75	921.82 ± 343.39
11	1130.04 ± 458.03	1045.86 ± 425.72
12	1290.72 ± 568.50	1183.12 ± 523.60
13	1469.79 ± 699.91	1334.70 ± 639.22
14	1669.0 ± 855.37	1501.83 ± 775.04*
15	1890.27 ± 1038.37*	1685.85 ± 933.81*

**P* value is not significant

^a^Alternative model: two part model regression analysis on the main model after adding control status

^b^2010 Canadian dollars

## Abbreviations

BC: British columbia
CAD: Canadian dollars
EBA study: Economic burden of asthma study
ED: Emergency department
GINA: Global initiative for asthma
NOC: National occupation classification
OR: Odds ratio
PDC: Proportion of days covered (by any asthma controller medication)
RR: Relative rate
SCQ: Self-administered comorbidity questionnaire
SD: Standard deviation
VOLP: Valuation of Lost Productivity
WPAI: Work productivity and activity impairment

## References

[1] Diederichs CP, Wellmann J, Bartels DB, Ellert U, Hoffmann W, Berger K (2012). How to weight chronic diseases in multimorbidity indices? Development of a new method on the basis of individual data from five population-based studies. J Clin Epidemiol.

[2] Starfield B, Kinder K (2011). Multimorbidity and its measurement. Health Policy Amst Neth.

[3] Fortin M, Lapointe L, Hudon C, Vanasse A (2005). Multimorbidity is common to family practice: is it commonly researched?. Can Fam Physician Médecin Fam Can.

[4] Ledford DK, Lockey RF (2013). Asthma and comorbidities. Curr Opin Allergy Clin Immunol.

[5] Prosser R, Carleton B, Smith A (2010). The comorbidity burden of the treated asthma patient population in British Columbia. Chronic Dis Can.

[6] Zhang T, Carleton BC, Prosser RJ, Smith AM (2009). The added burden of comorbidity in patients with asthma. J Asthma Off J Assoc Care Asthma.

[7] Patel MR, Janevic MR, Heeringa SG, Baptist AP, Clark NM (2013). An examination of adverse asthma outcomes in U.S. Adults with multiple morbidities. Ann Am Thorac Soc.

[8] Rank MA, Liesinger JT, Ziegenfuss JY, Branda ME, Lim KG, Yawn BP (2012). Asthma expenditures in the United States comparing 2004 to 2006 and 1996 to 1998. Am J Manag Care.

[9] Rank MA, Shah ND (2014). Multiple chronic conditions and asthma: implications for practice and research. J Allergy Clin Immunol Pract.

[10] Sumino K, O’Brian K, Bartle B, Au DH, Castro M, Lee TA (2014). Coexisting chronic conditions associated with mortality and morbidity in adult patients with asthma. J Asthma Off J Assoc Care Asthma.

[11] Cazzola M, Calzetta L, Bettoncelli G, Novelli L, Cricelli C, Rogliani P (2011). Asthma and comorbid medical illness. Eur Respir J.

[12] Steppuhn H, Langen U, Scheidt-Nave C, Keil T (2013). Major comorbid conditions in asthma and association with asthma-related hospitalizations and emergency department admissions in adults: results from the German National Health Telephone Interview Survey (GEDA) 2010. BMC Pulm Med.

[13] Brinke A t, Sterk PJ, Masclee A a M, Spinhoven P, Schmidt JT, Zwinderman AH (2005). Risk factors of frequent exacerbations in difficult-to-treat asthma. Eur Respir J.

[14] Doz M, Chouaid C, Com-Ruelle L, Calvo E, Brosa M, Robert J (2013). The association between asthma control, health care costs, and quality of life in France and Spain. BMC Pulm Med.

[15] Gershon AS, Wang C, Guan J, To T (2010). Burden of comorbidity in individuals with asthma. Thorax.

[16] Ehteshami-Afshar S, FitzGerald JM, Doyle-Waters MM, Sadatsafavi M (2016). The global economic burden of asthma and chronic obstructive pulmonary disease. Int J Tuberc Lung Dis.

[17] Sadatsafavi M, Rousseau R, Chen W, Zhang W, Lynd L, FitzGerald JM (2014). The preventable burden of productivity loss due to suboptimal asthma control: a population-based study. Chest.

[18] Chen W, Lynd LD, FitzGerald JM, Marra CA, Rousseau R, Sadatsafavi M (2015). The added effect of comorbidity on health-related quality of life in patients with asthma. Qual Life Res Int J Qual Life Asp Treat Care Rehab.

[19] Sangha O, Stucki G, Liang MH, Fossel AH, Katz JN (2003). The Self-Administered Comorbidity Questionnaire: a new method to assess comorbidity for clinical and health services research. Arthritis Rheum.

[20] Andreasson E, Svensson K, Berggren F (2003). PRP11 The validity of the work productivity and activity impairment questionnaire for patients with asthma (WPAIASTHMA): Results from a web-based study. Value Health.

[21] Zhang W, Bansback N, Boonen A, Severens JL, Anis AH (2012). Development of a Composite Questionnaire, the Valuation of Lost Productivity, to Value Productivity Losses: Application in Rheumatoid Arthritis. Value Health.

[22] Pauly MV, Nicholson S, Polsky D, Berger ML, Sharda C (2008). Valuing reductions in on-the-job illness: “presenteeism” from managerial and economic perspectives. Health Econ.

[23] Government of Canada E and SDC. National Occupational Classification [Internet]. 2013. Available from: http://www30.hrsdc.gc.ca/NOC/english/NOC/2006/Welcome.aspx. Accessed 10 Mar 2016.

[24] Mihaylova B, Briggs A, O’Hagan A, Thompson SG (2011). Review of statistical methods for analysing healthcare resources and costs. Health Econ.

[25] Yuan Y (2010). Multiple imputation for missing data: Concepts and new development (Version 9.0).

[26] Aubas C, Bourdin A, Aubas P, Gamez AS, Halimi L, Vachier I (2013). Role of comorbid conditions in asthma hospitalizations in the south of France. Allergy.

[27] Hakola R, Kauppi P, Leino T, Ojajärvi A, Pentti J, Oksanen T (2011). Persistent asthma, comorbid conditions and the risk of work disability: a prospective cohort study. Allergy.

[28] Moullec G, FitzGerald JM, Rousseau R, Chen W, Sadatsafavi M (2015). Economic Burden of Asthma (EBA) study team. Interaction effect of psychological distress and asthma control on productivity loss?. Eur Respir J.

